# MechoA+: A Chemical Structure Profiler Raising the Bar for the Prediction of Mechanisms of Toxic Action for Chemical Safety Assessment

**DOI:** 10.1021/acs.est.5c18657

**Published:** 2026-06-08

**Authors:** Gaspard Levet, Franklin J. Bauer, Paul C. Thomas, Mark T. D. Cronin, Jayne Roberts, Steve Gutsell, Bruno Campos, Geoff Hodges, James Firman

**Affiliations:** † KREATiS SAS, 23 rue du Creuzat, ZAC de St-Hubert, 38080 L’Isle d’Abeau, France; ‡ School of Pharmacy and Biomolecular Sciences, 4589 Liverpool John Moores University, Byrom Street, Liverpool L3 3AF, U.K.; § Safety, Environmental and Regulatory Science (SERS), Unilever, Colworth Science Park, Sharnbrook, Bedfordshire MK44 1LQ, U.K.

**Keywords:** (eco)toxicity, structure-activtiy relationship (SAR), mechanism of action, molecular initiating event (MIE), new approach methodologies (NAMs), structural profilers

## Abstract

With stakeholders in chemical regulation increasingly advocating for nonanimal testing methodologies, there is a need for reliable *in silico* tools with wide applicability domains to predict both environmental and human hazards. In this context, the *in silico* structure-based MechoA+ scheme has been developed to predict molecular initiating events, appropriate for both mammalian and ecotoxicity by merging and refining two previous classification models, MechoA and Sapounidou–Firman schemes. The resulting model is a new decision tree composed of 152 structural alerts able to classify a wide range of substances within 6 mechanistic classes and 27 subclasses and two rules excluding substances out of the scope of the scheme. Analysis of MechoA+ scheme predictions shows a higher percentage of valid predictions (92% predicted positive value on the training set) and wider structural, mechanistic, and taxonomic domains than the previous models on a data set of more than 70,000 substances (covering cosmetics, pesticides, etc.), achieving predictions for 80% of substances. Since MechoA+ is implemented within readily available software tools, its widespread adoption will facilitate more accurate hazard assessments, QSAR building, read-across, and grouping, strengthen regulatory decision-making, and support safer chemical design in early-stage research and development.

## Graphical abstract

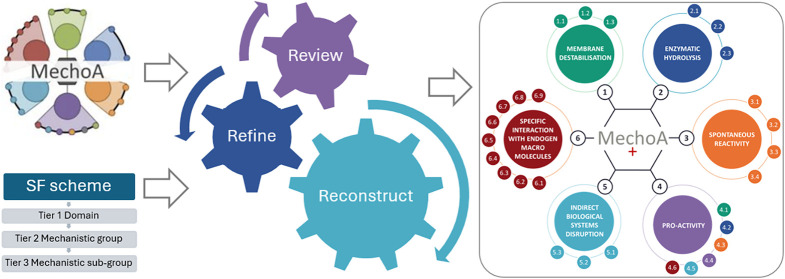

## 1 Introduction

In Europe and North America, up to 100,000 substances are registered with 40,000–60,000 estimated to be used routinely.^1–4^ Yet, only a relatively small proportion of these have sufficient data available to support robust safety evaluations, particularly regarding acute toxicity and exposure. Among the promising tools to address this shortfall, computational approaches can contribute hazard insights. New approach methodologies (NAMs), including *in silico*, are advancing rapidly and have great potential to hasten efficient and high-quality data generation following the 3Rs principles for animal testing (replacement, reduction, and refinement) while concomitantly reflecting public expectations for more ethical science.^5–7^

With the advent of the adverse outcome pathway (AOP) concept^8^ (Figure 1), authorities increasingly consider NAMs as a means to provide mechanistic evidence and support chemical safety assessment.

**Figure 1:**
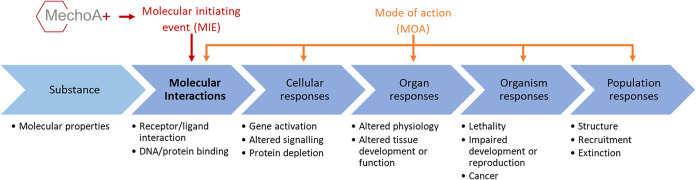
Adverse outcome pathway (AOP) scheme (adapted from Ankley et al.^8^).

Computational tools that apply AOP principles are of utmost importance, providing additional understanding of the mechanisms behind toxicity, something that *in vivo* regulatory studies often fail to do. This effectively supports both human health toxicologists and ecotoxicologists in their hazard assessment, helping regulatory authorities to prioritize groups of substances of concern, supporting read-across strategies, aiding the early or late-stage development of substances (safe and sustainable by design (SSbD) strategies^9^), and also drawing cross-species extrapolations.

The last few decades have seen progress in tools for screening, prioritization, and toxicological profiling based on structural alerts. Initially, expert-based mode of action (MoA) schemes were developed^10–14^ and machine learning methods were also applied to classify MoAs for aquatic organisms, e.g., MOATox.^15^ However, traditional MoA-based schemes do not cover a very large chemical space, limiting their applicability domain (AD), and they were mostly focused on a limited number of ecotoxicological end points (notably acute fish tests).^16^ Furthermore, they do not systematically refer to molecular initiating events (MIEs) but are often more related to the observed adverse effect on an organism or a population. Such observations can result from different key events downstream of the MIE leading to confusion.^16,17^ In such cases, the MIE is not always known or understood, and the mechanistic interpretation may be lacking to correctly interpret the results. Similar efforts in the field of mammalian toxicology (aiming at human health) are mostly end point-specific.^18–21^

To address these issues, classification tools focusing on the MIE, also called mechanistic schemes, started to emerge. Overall, distinctions between the first-generation (MoA-based) schemes and second-generation (mechanistic) schemes were discussed by Firman et al.^22^ in 2022.

While MIE-based profilers were developed earlier for specific end points^23–25^ or chemical classes,^26^ in 2017, Bauer et al.^27,28^ developed a structure-based profiler (or more formally a structure–activity relationships (SAR) model) called MechoA (standing for mechanisms of toxic action) scheme which covered a wider range of mechanisms, chemicals, and species. This scheme assigns organic monoconstituent substances into six broad mechanistic classes, further divided into 23 subclasses (Figure S1), based on both literature review and toxicological data analysis. The output of the model, called the MechoA of the substance, is more than just the MIE. It includes the MIE, the taxon with which it is associated, as well as subsequent key events (such as mutagenicity or growth impairment, etc.) when possible. To effectively predict mechanisms of toxic action for substances that can match multiple alerts, the MechoA scheme uses a decision tree to prioritize the primary MIE(s) which will ultimately lead to the downstream effects observed *in vivo*.

The MechoA scheme had several advantages over previous schemes, as the applicability domain (comprising mechanistic and structural domains) was extensive. In addition, the scheme was structured such that the world of organic chemistry could be separated into a limited number of classes and subclasses, and the alerts were structurally prioritized. Furthermore, the scheme has been regularly updated since its initial publication. It is included as the “iSafeRat Mechanisms of Toxic Action profiler” within the OECD QSAR Toolbox^29^ as a downloadable plug-in and has also been implemented in iSafeRat Desktop software,^30^ where it provides automated results. Furthermore, the QSAR producer, KREATiS, had already made efforts to directly link MechoAs to specific (eco)­toxicity QSARs. Finally, compared to other more end point-specific profilers, attention was paid to unifying the, until now separate, languages used in toxicology and ecotoxicology and integrating them into the same model.

A similar concept to the MechoA scheme was published by Sapounidou et al.^31^ and further improved by Firman et al.,^22^ hereafter referred to as the “Sapounidou–Firman” (SF) scheme. The SF scheme^22,31^ categorizes compounds into 3 broad mechanistic classes, so-called domain (tier 1), with two further levels of subclasses: the “mechanistic group” (tier 2:10 classes) and the “mechanism” subgroup (tier 3:25 classes) (Table S1), based on a literature review. Furthermore, the scheme is available as a KNIME workflow, with each alert coded with SMILES Arbitrary Target Specification (SMARTS)^32^ strings such that the SF scheme can be applied in batch by KNIME users, and integrated into larger workflows, e.g., for risk assessment purposes. If several alerts are identified for a compound, all the alerts that have been matched are given by the tool and no prioritization rules are defined. More details on outputs of the scheme can be found in Figure S2. Concerning the structural space, the tool covers mostly organic chemistry, similarly to the MechoA scheme. However, it includes more biocidal and pharmaceutical actives than MechoA, as well as some organometals. The applicability of the scheme to several areas of chemistry has been extensively described by Firman et al.22. Overall, the SF scheme also has a wide applicability domain (comprising mechanistic and structural domains). The scheme is automated and, at the time of publication, is in the process of being integrated into the OECD QSAR Toolbox^29^ as a downloadable plug-in.

To leverage and extend the strengths of both MechoA and SF schemes, the two have been merged into a single model called MechoA+ and further refined while maintaining the original MechoA classification structure. This decision was justified by the existing prioritization scheme that was existing in the MechoA scheme but not in the SF scheme. The resulting MechoA+ scheme showed better predictive capacity and a wider and better-defined applicability domain. The main goal of the work presented here is to provide an *in silico* tool capable of assigning molecules to a probable mechanism of (eco)­toxicological action (MechoA). This tool enables a more mechanistically driven regulatory paradigm, where appropriate hazard assessment strategies can be applied based on the predicted MechoA of substances with interpretable results. Here, we compare the new and existing schemes and assess the respective outputs of each tool.

## 2 Materials and Methods

### 2.1 Structural Alert Construction

The methodology used to build MechoA+ was similar to that used for the construction of MechoA and SF schemes. It is based on previous knowledge with an additional literature search in scientific literature, databases, and tools (e.g., EFSA,^33^ ECHA,^34^ IARC,^35^ PubChem,^36^ DrugBank,^37^ OECD QSAR Toolbox, etc.).

The initial step in merging both tools into MechoA+ involved a comprehensive review of each alert from previous schemes. It included an in-depth analysis and examination of available literature and experimental studies to validate the putative mechanisms and identify observed effects expected from such MIEs in experimental studies. The presence or absence of a toxicological effect, when well-documented and linked to a biological response, provided evidence to confirm the MIE for a chemical or its family. The goal was to enhance the understanding of the intrinsic mechanisms for each substance, clarify the applicability domain of each alert, and describe the taxa (i.e., biological species) impacted by the MIE.

During the iterative process of reviewing all alerts and associated mechanisms, shared alerts between both schemes were fused, and their structural features were reviewed. Alerts not shared were amended, if necessary, and incorporated into MechoA+. The scope of alerts from previous schemes (both structural and taxonomic) was either restricted or extended on the basis of the literature review and toxicological data analysis. Additionally, some alerts were reallocated to different subclasses or removed upon review of further evidence.

To build MechoA+ alerts, the identification of common substructures between the analogues is necessary. In this paper, we define the term “analogue” as a molecule bearing a similar molecular pattern or functional group to a prototypical chemical substance or group, allowing the molecule to interact with the same biological target. Analogues related to each alert with their corresponding MIEs were collated and a training set/internal validation set was built (further described below). The alerts were built based on evidence from the literature, the list of analogues and chemical knowledge. Since most alerts are based on less than 25 analogues, large language models or other techniques which typically require extensive data sets were not used. For alerts related to direct interaction with a specific biological target in an organism (e.g., receptors, ion channels, etc.), the taxonomic applicability was refined by using the protein ortholog database EggNOG v5.0.0 ^38^ followed by use of the Taxonomy Browser from NCBI^39^ (Note S1). The scheme was developed mostly for regulatory applications; therefore, there is an emphasis on more regulatory applicable taxa such as fish, daphnids, mammals, and plants (including unicellular algae). However, other taxa may also be described (e.g., bacteria, humans).

### 2.2 Prediction Results Formatting

The MechoA format was refined during the project, with specific text allocated to mechanism classes, subclasses, and taxonomic identifications. The format used is ″MechoA xxY.Z”, where ″xx″ represents the taxa code, ″Y″ the mechanism class, and ″Z″ the mechanism subclass. A full description of text codes used to describe outputs is given in Note S2. Each prediction includes a descriptive text explaining the mechanism shortly and its applicable species, often detailing subsequent key events. For those looking for more information and bibliographic sources, the profiler output is linked with the MechoApedia^40^ webpage that describes the MIEs and additional key events in more detail.

### 2.3 Implementation of the Alerts in iSafeRat Desktop

Given the challenges associated with describing particular chemical patterns (see Note S3), when seeking to implement and automate alerts, the authors used a combination of SMARTS strings (through the RDKit library^41^) and proprietary C++ code developed by KREATiS based upon a molecular connectivity matrix deduced from the input SMILES code. Additionally, to read some unconventional SMILES, some transformation functions have been implemented.

### 2.4 Decision Tree

MechoA+ scheme is a linear decision tree of a set of 154 rules, or “alerts,” detailed in Table S2. A simplified representation of the decision tree is shown in Figure 2.

**Figure 2:**
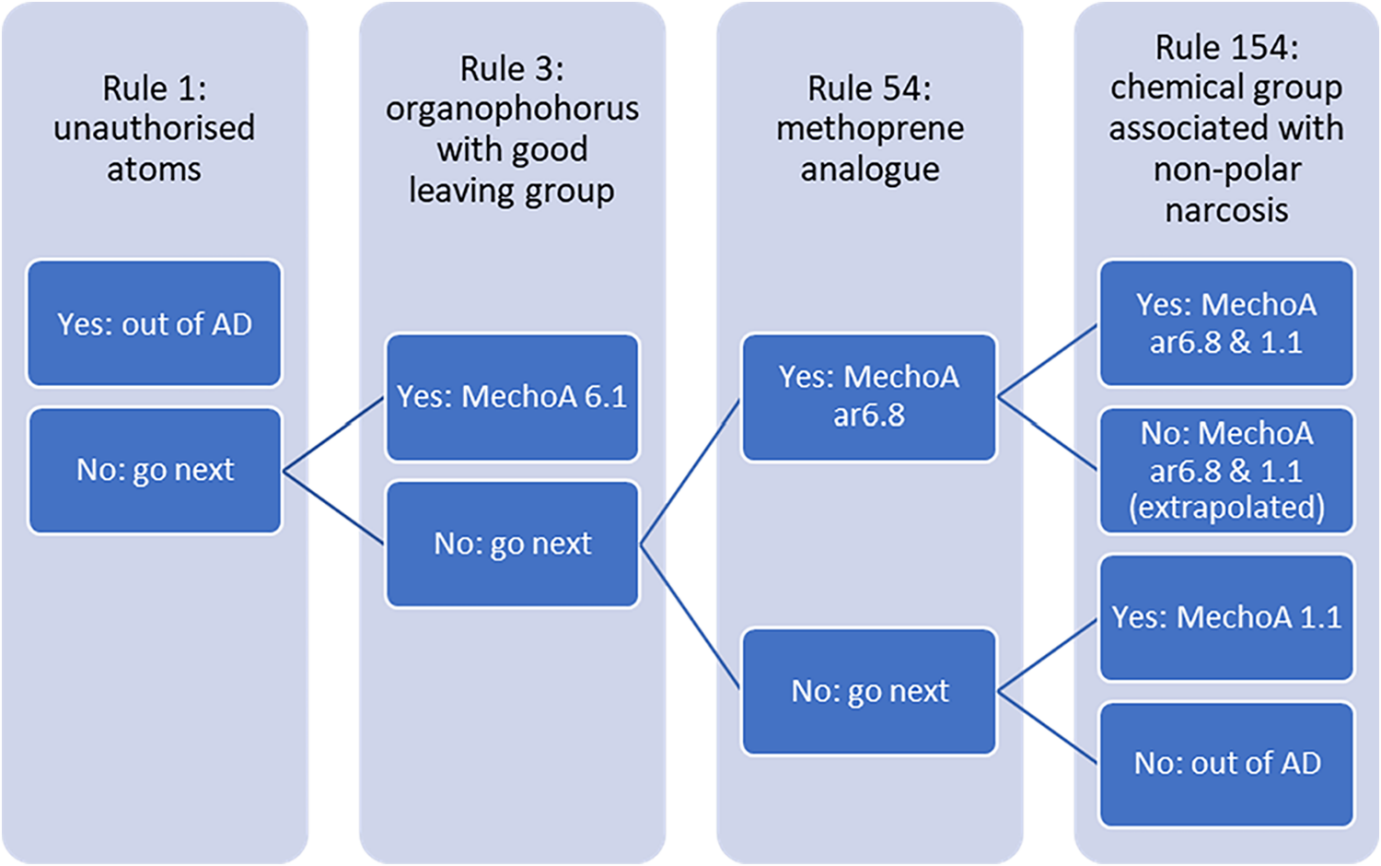
Simplified representation of MechoA+ decision tree.

At first, an exclusion rule (rule 1) is implemented, filtering only authorized atoms, forbidding the presence of inorganics or mixtures. Then, the software evaluates each rule sequentially, and the decision tree continues until alerts are matched across all relevant taxa. Alert number 150 addresses substances outside the applicability domain, reducing the derivation of false-negative or false-positive results, notably for the remaining 4 rules. Once alert number 154 is reached, the substance will be predicted with MechoA 1.1 (nonpolar narcosis) in addition to any previously identified MechoA, unless no chemical groups known to be associated with nonpolar narcosis are present and no other MechoA could be attributed by other rules of the tree.

Similar to the MechoA scheme, the decision tree of MechoA+ prioritizes the alerts based on the toxicity potential of the substances. The goal in simple terms is to sort the alerts in the order from the most to least potent mechanisms of toxicity. This ranking of alerts was done using both expert knowledge based on literature-derived toxicological data analysis and further by the examination of structural domain overlap between alerts. Once a MechoA is predicted for all species, alerts further down the decision tree are not run. Therefore, the output of the tool focuses on the most relevant alerts for each substance, avoiding an excess of information for users.

### 2.5 Training Set/Internal Validation Set

A training set comprising 2,091 substances, detailed in Table S3, was assembled from various sources, among which are cosmetic products, antibacterial, poisons, pesticides, drugs, etc. For each substance, available identification data such as SMILES notation, names, PubChem CIDs, and CAS numbers were collected, and one or several alert numbers were associated. Additionally, mechanistic evidence and available (eco)­toxicological information supporting each alert were evaluated for plausibility. This evaluation included theoretical chemical and biological knowledge, apical end points, and in vivo and in vitro data. The data set presents this weight of evidence. It includes the literature references, the scheme predictions from the current model version, and additional information on model evaluation.

In short, the validation process is 2-fold: assessing ″Experimental alert validity,″ which questions whether the experimental and literature evidence matches the MechoA expected from the alert, and ″Prediction alert validity″, which evaluates whether the substance is predicted by the software according to the expected alert. Based on these two validation criteria and on the results of multiple alerts potentially matched, an “Overall validity” is determined (for more explanation, see Table S4 & S5, Note S4). Finally, the performance of each alert was calculated based on the training set. To account for the underlying uncertainties associated with each prediction, results are not divided into “valid” (equivalent to “True positive”) or “invalid” (equivalent to “false positive”) only, but an additional “partially valid” result was considered if only part of the predicted MechoA matches experimental evidence, or conversely. Further details on statistics are given in the results section.

### 2.6 Analysis of MechoA+ Scheme and Comparison with Former Schemes

The coverage of structural and mechanistic space of the chemical classification scheme implemented in iSafeRat Desktop has been analyzed using two separated data sets.

First, the training set of 2,091 chemicals having an experimentally known mechanism (Table S6) was used.

Second, an external set, hereon called “test set,” of 76,120 substances, retreived from Firman et al.^22^ Substances were classified broadly as a function of the inventories from which they were sourced: REACH preregistration list, pharmaceuticals (i.e., DrugBank & Pharma), cosmetic constituents (COSMOS), pesticides, and botanical extracts (Table S7). Only their SMILES annotation and inventory source are known (SMILES can be found in Table S8).

A comparison was made of the combined alert results from the MechoA+ scheme with those from the SF and MechoA schemes, aligning and converting the classes and subclasses to facilitate comparison. The alignment of each subclass comparing MechoA v2.2 as implemented in the OECD QSAR Toolbox, the SF scheme (latest version), and MechoA+ v1.0 is presented in Table S9. To present a meaningful and understandable comparison for each substance, only mechanism classes and not subclasses were compared, and duplicate results were removed, e.g., a substance predicted subclass 3.1 and 3.4 was converted to a single class 3 result, and a substance predicted subclass 6.5 and 2.2 was converted to results class 2 and class 6. Aligned predictions and MechoA, MechoA+, and SF scheme output are presented in Tables S6, S8, S10, and S11.

A t-distributed stochastic neighbor embedding (t-SNE) visualization^42^ based on MACCSKeys fingerprint^41^ was used to assess and compare the chemical structural coverage of the models.

## 3 Results and Discussion

### 3.1 MechoA+ Classification

The development of MechoA+ involved a detailed examination of the similarities and differences between the MechoA and SF schemes. While they exhibit clear mechanistic overlaps, they differ in how they define classes, structural alerts, and the extent of mechanistic and chemical coverage, leading to partial overlaps and unique gaps in each scheme (Figure S3). Because their coverage is more complementary rather than redundant, merging them into a unified MechoA+ scheme substantially expanded the structural, mechanistic, and taxonomical applicability. The MechoA scheme architecture was chosen as the foundation for integrating SF scheme alerts into the new MechoA+ scheme, since the MechoA scheme had a broader mechanistic classification (Figure 3).

**Figure 3:**
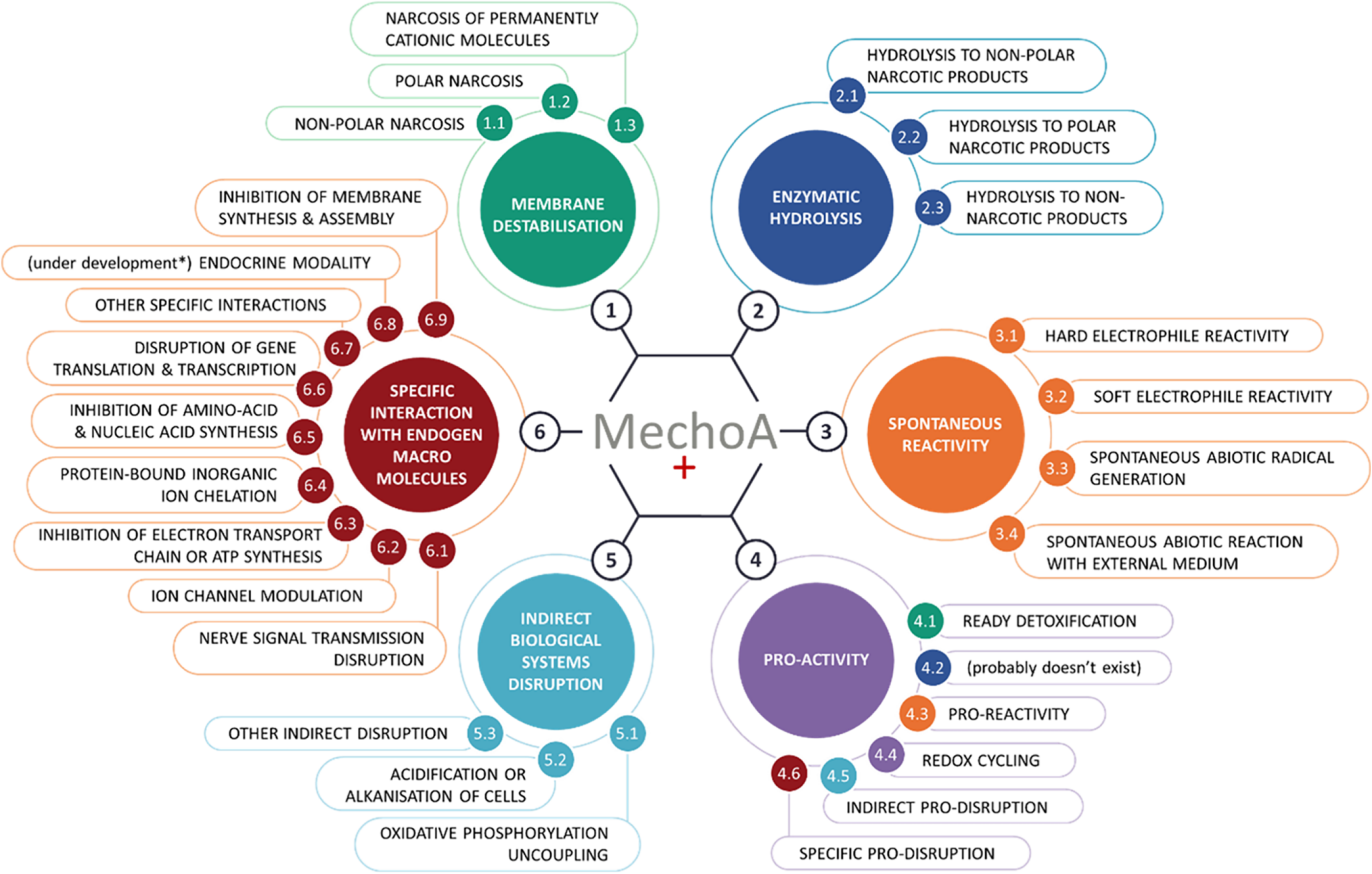
MechoA+ wheel presenting general classes and subclasses.

Note: subclass 4.2 is concerned with substances that would be metabolized into a hydrolyzable product behaving with a MechoA 2, while the parent itself would not be hydrolyzable. However, to the knowledge of the authors, such a situation does not happen, and it was thus decided to omit this subclass of class 4.

MechoA+ retains the six general classes from MechoA with slight change in naming: CLASS 1: membrane destabilization which concerns mostly narcotic substances, CLASS 2: enzymatic hydrolysis related to ester, carbonate, or phosphates substances mostly, CLASS 3: spontaneous reactivity, often related to protein or DNA adduct formation in a nonspecific way, CLASS 4: proactivity, for substances for which their metabolites is related to toxic mechanism of action, except with class 4.1 where detoxification is considered, CLASS 5: indirect biological systems disruption, ranging, for example, from the production of reactive oxygen species (ROS) to indirect disruption of cells because of proton gradient change in its environment, and CLASS 6: specific interaction with endogenous macromolecules, for instance MIE that are often found for pharmaceuticals or biocides.

It now contains 27 subclasses to accommodate additional molecular initiating event (MIE) descriptions. This is an expansion from the 23 subclasses in MechoA and the 25 (equivalent to “Tier 3 Mechanistic subgroup”) in the SF scheme. By refining and expanding its subclasses, MechoA+ successfully incorporates all SF and MechoA alerts.

The SF^22^ and MechoA^28^ schemes contain 183 and 69 alerts, respectively. This lower number in the MechoA scheme reflects the fact that structural features leading to the same MIE were often grouped into a single alert or a limited number of alerts. Each scheme may have different alerts for the same class or subclass, resulting in different mechanistic and structural domains despite some commonalities. Thus, for every alert of the SF scheme and MechoA scheme that corresponds to a similar MIE, they were combined in MechoA+ under one unique alert where possible. Where alerts from the two schemes were in contradiction, the underlying weight of evidence was reviewed, and MechoA+ alerts were designed correspondingly.

The MechoA+ scheme has 154 rules, including 2 exclusion rules specifically designed for applicability domain restriction purposes. Thus, 152 MIE alerts were validated during the modeling process, for which one or several MIEs are attributed with a specific taxa applicability. All those alerts as well as the linear decision tree are presented in Table S2.

### 3.2 Applicability of the Prediction Across Taxa

An additional benefit of the new MechoA+ scheme over the individual MechoA and SF schemes is the increase in and better definition of taxonomical coverage. Indeed, these schemes were more focused on MIEs described for aquatic species. The taxonomical applicability of the MechoA+ scheme varies between alerts, depending upon the presence of the biological target and the ability of a species to metabolize the represented chemical structures, etc. Two methodologies were used to extrapolate the range of taxa in which an MIE is potentially susceptible to occur, depending upon whether the alert is considered “nonspecific” (61 alerts, 19 MechoA subclasses) or “specific” (91 alerts, 10 MechoA subclasses).

#### 3.2.1 Nonspecific MIEs

Nonspecific MIEs apply across all species, with exceptions whereby a metabolic (de)­activation can occur. These include mechanisms such as narcosis which, as the “baseline toxicity,” affects all species by disrupting cell membranes. Mechanisms involving reactivity (e.g., covalent binding to DNA/proteins), and indirect enzyme disruption (e.g., oxidative phosphorylation uncoupling) also affect all species. Nonspecific mechanisms impact commonly shared biological systems, therefore MechoA+ classes 1, 3, and 5 are considered nonspecific. For these nonspecific MIEs, taxonomic applicability was set to “all species”, unless (eco)­toxicological data show specific metabolism in certain species, leading to significant metabolites with different MechoAs.

#### 3.2.2 Specific MIEs

Molecules which are known to specifically target one or several biological molecules before or after metabolism enter the category of “specific” mechanisms. Inevitably, the taxonomic applicability of such alerts is more restrictive compared to alerts for nonspecific mechanisms, since the MIE is related to the presence of a specific target (e.g., receptor, enzyme, etc.) in the organism.

To achieve this, a rapid cross-species search methodology with EggNOG was developed (Note S1), refining the taxonomic applicability of such alerts compared to the MechoA or SF scheme. This new approach allowed us to exclude taxa that cannot be impacted by a specific MIE (such as a chemical acting on GABAergic chloride channels in animals but not in plants due to the absence of the target) while allowing the inclusion of additional taxa to those which have (eco)­toxicological data, if these taxa appear to have the same target. This method distinguishes MechoA+ from previous schemes, representing a significant step forward to better characterize the taxonomic applicability domain of each alert.

### 3.3 Training Set and Validation of the Alerts

Despite the challenges to generate statistics on such a model and data set, efforts were made to evaluate the quality of the alerts and thus the quality of the prediction results (Note S4). The variability and uncertainty of the results were taken into account with four different metrics: alert validity compared to experimental evidence, the prediction validity, and the overall validity of MechoA+ predictions.

Experimentally evidenced mechanisms, references, alert numbers, and predictions expected for each substance were compiled for the training set composed of 2091 substances (Table S3). Compared to the MechoA scheme with 491 substances in its training/validation set, and SF scheme, where substances associated with MIE were not explicitly described, though available through literature, MechoA+ work took into consideration a substantial amount of additional molecules to define a more comprehensive training set. This extended structural and toxicological information helped the authors refine restrictions for MechoA+ rules. MechoA+ AD rules defined in section 3.5 were implemented to face some limitations observed while validating the alerts in order to avoid the prediction of too many false positives especially for rules targeting a wide diversity of substances. Since it would not be possible to run bootstrapping or cross-validation methods on such an expert-based model, internal validation was done on the whole training set of MechoA+ “manually.”

### 3.4 Goodness-of-Fit of the Model (Sensitivity/Specificity Analysis)

The training set with 2091 substances, also used as an internal validation set, was created to assess the goodness-of-fit of MechoA+. Among these, a total of 1383 can be considered “valid,” or true positives (TP), meaning that the experimental weight of evidence gathered to justify the MIE(s) for these substances was conclusive and matched the predictions. “Invalid” predictions, or false positives (FP), were identified for 119 substances. Given the higher uncertainty associated with experimental evidence to ascertain the predictions, the remaining 589 substances could not be classified as either TP or FP, and these results were expressed as “Valid a priori,” “Partially valid,” “Partially valid a priori” or “Invalid a priori”, as explained in the Table S5 & S6 and Note S4.

The combined results of the “Overall validity” are presented in Table 1 below:

**Table 1:** Overall Scheme Validation

total number of substances	valid(TP)	valid a priori	partially valid	partially valid a priori	invalid (FP)	invalid a priori
2091	1383	332	234	8	119	15
100%	66%	16%	11%	0%	6%	1%
	82%		12%		6%	

Thus, on 1502 substances with a “clear” outcome in the training set (1383 Valid, 119 Invalid), 92% may be considered well predicted (TP) and 8% wrongly assigned (FP).

Additional data were collated during the course of the study for alerts that had limited supporting evidence in the initial training set to improve TP predictions. Compared to the previous models, this work provides more information to understand the reliability behind the given alerts or MechoA predictions. From the statistics of the training set (Table S2), it is possible to see that confidence in the prediction is largely alert-dependent. In general, for SARs, confidence in an alert increases when it is supported by a larger number of substances for which the prediction of the alert matches experimental observations. It is mostly the sensitivity of the alerts that is important since, in most cases, alerts were produced to predict the presence of a mechanism of toxicity rather than the absence of such mechanisms. Indeed, it is difficult to ascertain the absence of a given MIE. For MechoA+, increasing the number of analogues supporting a TP result increases the confidence that can be attributed to a given alert. However, adding substances without sufficient data decreases the percentage of TP, reducing the confidence in alerts. Conversely, alerts based on a few substances but with well-evidenced mechanisms can be considered as having higher confidence, especially if the structural features coded for the alert are much restricted to forbid substituents of unknown effect on the molecule. This is in general the case (few analogues but with well-evidenced mechanism) for all alerts concerning MIE, which are classified in subclasses 4.1, 4.5, 4.6, 5.1, 5.3, and the class 6. Additionally, alerts with less restriction in the structural patterns have higher prediction uncertainty due to potential fragments that could be outside of the intended scope of the alert.

Currently, the training/internal validation set provides an overview of each alert’s performance but is limited for assessing overall goodness-of-fit. Future improvements could include additional statistical evaluations and reliability indicators based on the training set.

### 3.5 Evaluation of the Predictivity

An external validation set would also enhance model evaluation, although the scarcity of mechanistic toxicological data for substances in the domain of all alerts of the present scheme makes the constitution of such an external test set difficult. Nevertheless, several independent approaches encompassing either direct or indirect evidence provide insights into the predictivity of the model.

Indirect evidence is provided by the existing iSafeRat models for acute and chronic toxicity to fish, daphnia, and algae, which are a set of linear regressions between aquatic ecotoxicity and subcooled liquid water solubility values. One regression per mechanistic class is used for each species and each end point. Each mechanistic QSAR is built from rigorously validated experimental water solubility and toxicity data, and the regression is statistically evaluated. Each regression is composed of mixed chemical structures related to a single MechoA+ subclass. All experimental values of the training and external validation set typically fall within a factor of 3 of the predicted value of the model regression (as reproducible as experimental data itself). While such models do not yet exist for all subclasses, a wide range of them, encompassing the majority of general and specialty chemical classes, are covered by the QSARs, especially subclasses 1.1, 1.2, 1.3, 2.1, 3.1, 3.2, 4.4, 5.2, 1.2&5.2 and anC4.3&1.2. This set of different ecotoxicity models has been statistically validated (further details provided in the QSAR Model Reporting Format (QMRF)^43,44^ ). These QSARs present a well-established body of evidence that a significant part of the mechanistic classes described in MechoA+ are valid, at least for algae, daphnids, and fish. Of note, some substances from the training and external validation sets of these iSafeRat models are present in the training set of MechoA+ (however, for intellectual property reasons, the data sets of these models cannot be disclosed here) (Note S5).

Additionally, the recent MOA-based data set of Kramer et al.^45^ is one example that could be used for additional future partial evaluation. As was observed for ecotoxicity QSARs, this data set will also not encompass the complete scope of taxonomical and mechanistic coverage of MechoA+ for a complete predictivity assessment. However, building on further independent additional evidence will help understand the versatility, limits, and power of the predicted mechanistic tool.

### 3.6 Decision Tree

Sets of priority rules between alerts are defined for MechoA+ as a function of the “expected acute toxicity potential” resulting from the associated mechanisms of toxicity. In general, alerts are organized with the following order, depending on the MechoA predicted by the alert: first exclusion rule > MechoAs 6.1, 6.2, 6.3 > MechoAs 3 > other MechoAs 6 > MechoAs 5 > MechoAs 4, 2.2, 2.3 > MechoA 1.3, 1.2 > second exclusion rule > MechoA 2.1 > MechoA 1.1 > Out of applicability domain (AD).

While this means that complementary MIEs may be hidden while running the prediction, the approach aims to reduce the risk of misinterpretation by the user and ease the formation of a category for reglementary purpose. Contrarily to the SF scheme, the idea of MechoA+ was to provide only one response per taxon using a prioritization tree, easing interpretation of the results of the model. For example, if a substance has both the ability to be an acetylcholinesterase (AChE) inhibitor and a hard electrophile giving adducts to proteins, MechoA would only report it as an AChE inhibitor, which would not be predictive of a potential skin sensitization effect due to the adduct formation (more about this in the next section).

To be outside of AD of MechoA+, a molecule is either targeted by the exclusion rules of alert 1, alert 150, or no alert is found for a substance after searching through all of the alerts of the decision tree.

With this methodology, it is possible to make a prediction for a variety of substances, maintaining the very large possibility of combination of alerts while still limiting the model to “only” structures that should be known by the scheme.

While this model has been developed using a manually designed decision tree, machine learning methods (such as XGBoost, Random forest, and TabPFN-2.5) may be considered for future updates to the scheme in order to compare the results with our expert-based model MechoA+.

### 3.7 Diversity of Results

A wide diversity of MechoA predictions can be obtained when several MIEs are identified for the same substance. For example, this would be the case when an MIE is only related to a particular species. The examples below (Table 2) highlight this diversity.

**Table 2:**
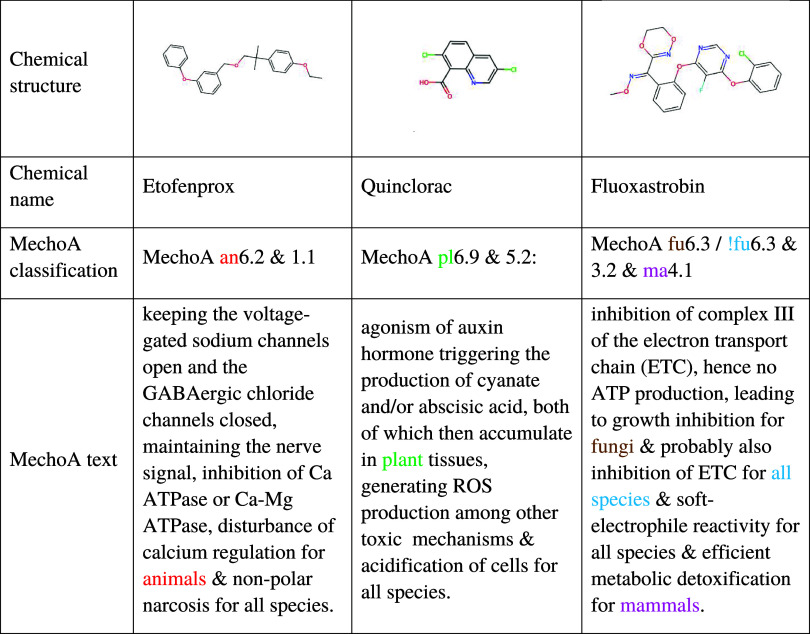
Examples of MechoA+ Predictions Showing Several Species-specific MIEs In the MechoA Classification: Absence of Taxa Codes: Means All Species, ‘!’: Means All Species Excluding the Following^a^

a See Note S2 for a full explanation of the classification nomenclature.

Depending on the end points of interest for the user, all or part of the information provided in a prediction may be useful. Also, part of the information may be missing because of the decision tree strategy (see section above), i.e., for substances identified to have interactions with several biological targets for the same species.

Future refinements may introduce a feature allowing advanced users to choose between predictions with alert prioritization or not (i.e., all alerts would be run). While this would require further expansion of (eco)­toxicological knowledge and refinement of the alerts and the decision tree, it would enhance the tool’s versatility.

### 3.8 Coverage

#### 3.8.1 Coverage of Chemicals within the Training Set and Comparison with Former Schemes

The MechoA+ classification scheme is able to classify 95% (1991 out of 2091) of substances in its training set (Figure 4 and Table S12). The remaining 5% were not classified either because they were out of the applicability domain or because the software could not read the corresponding SMILES. On another note, on the training set of MechoA+, both the MechoA scheme as well as SF scheme have a lower prediction rate. This was not surprising given that MechoA+ is the result of merging alerts from both schemes, and most specifically alerts that could be found in only one of them.

**Figure 4:**
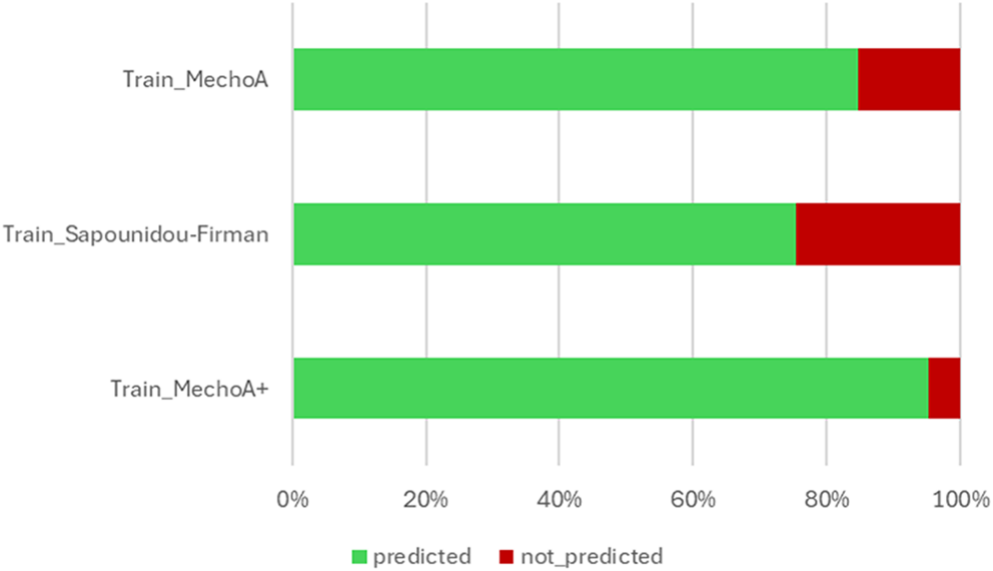
Comparing the percentage of predicted substances for each scheme based on the training set (2091 substances).

In the majority of cases, substances were purposely out of the domain owing to the decision tree of the model. Indeed, 4% triggered the first exclusion rule of the model (alert 1). This underlined the need for such a rule at the beginning of the decision tree, because overall those substances either are mixtures, inorganics, or else contain wrongly annotated SMILES.

To further describe classification results of the 1383 substances considered TP (see Section 3.4), the results obtained by MechoA+ and the two former schemes (MechoA and SF) in each class were compared and are presented in Figure 5.

**Figure 5:**
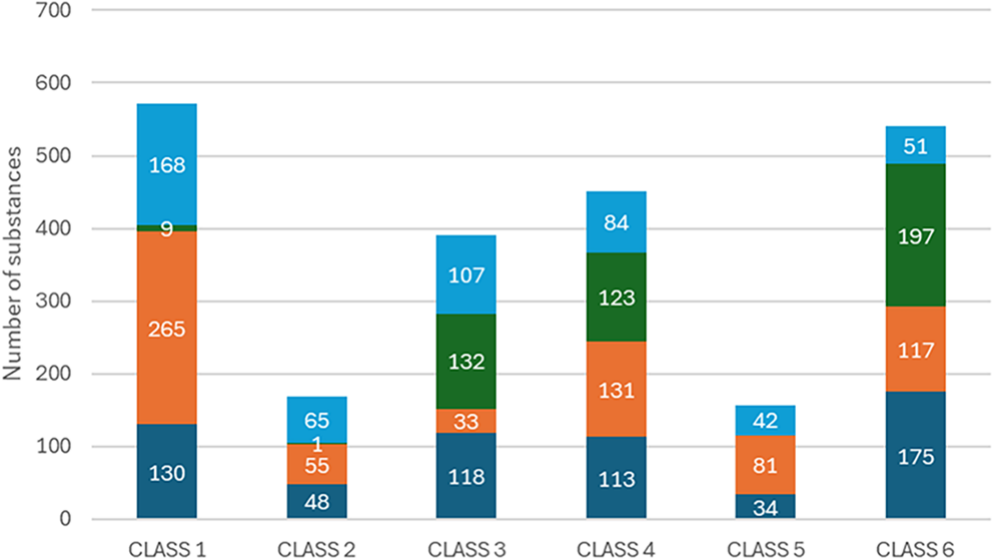
Number of true positive predictions obtained for each MechoA+ classes. Multiple occurrences of the same class number in a single prediction were counted as a single occurrence. Dark blue: substances that are TP for all 3 schemes simultaneously; Orange: substances that are TP for MechoA and MechoA+ but not SF; Green: substances that are TP for SF and MechoA+ but not MechoA; Light blue: substances that are TP for MechoA+ alone.

The results indicate that the SF scheme has contributed greatly to alerts developed in class 3, class 4, and class 6. As to the MechoA scheme, it contributed significantly in each class, especially classes 1, 2, and 5. The additional contribution of the alert refinements performed in the MechoA+ project is also evidenced in Figure 5, showing that MechoA+ is a significant improvement over the mere combination of previous schemes. Taxonomic attribution of each prediction was not taken into account in the preliminary analysis. Future comparative work with an external test set would further increase our understanding of overall predictivity.

#### 3.8.2 Coverage of Chemicals within an Extended Inventory and Comparison with Former Schemes

##### 3.8.2.1 Domain Stretch

A “test” set of 76,120 compounds coming from Firman et al.^22^ was used to evaluate the coverage of MechoA+ (Table S12). A classification could be achieved for 80% of the substances (60,684 substances). Eight percent were considered out of applicability domain (corresponding to alert 1 and 150 of the decision tree). Three percent of substances were identified as having a chemical group not correctly detected (corresponding to alert 150 of the decision tree). Nine percent could not be read by the software because the SMILES format was not compatible with iSafeRat Desktop.

##### 3.8.2.2 Coverage in Function of Chemical Uses

This test set is the combination of various databases (see **2.6**), which cover a broad variety of chemical structures and their uses. At least ∼ 80% of substances in the test could be predicted using MechoA+ within each database covering a particular use (Figure 6). Thus, the MechoA+ scheme covers a large spectrum of chemistry uses, rendering it highly suitable for use in any kind of chemical application in order to gather mechanistic information at a fast pace.

**Figure 6:**
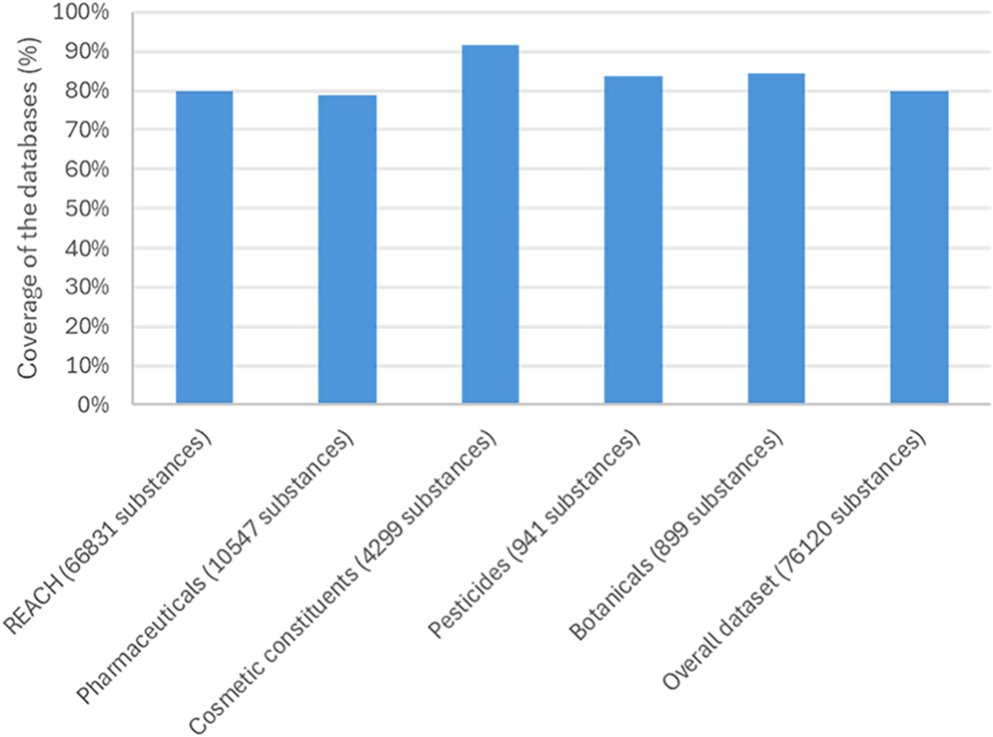
Percentage of substances predicted with the MechoA+ scheme for each type of use.

Regarding the coverage per database (Figure 6), MechoA+ covers cosmetic constituents more than any other substances (about 90%), while pharmaceuticals have the lowest coverage (79%). For pharmaceutical databases (DrugBank and Pharma), it should be noted that they not only include biological active substances but also the formulation products used as ingredients or metabolites. Thus, the real proportion of biologically active pharmaceuticals predicted may well be lower than 79%. Given the number of pharmaceutical products in this database, it is not possible to verify if the MechoA+ prediction covers the specific MIE defined in the pharmaceutical application or an off-target mechanism, which would probably be more pertinent in assessing the toxicity of substances for nontarget species. To verify and further improve the quality of the prediction for biologically active substances, it will be necessary to augment the training set in the future with pharmaceutical substances with validated mechanistic data. Overall, the scheme demonstrated consistent performance across diverse data sets.

### 3.9 Analysis of the Classification Results

The results of the affiliation with classes were studied for each scheme (Figure 7).

**Figure 7:**
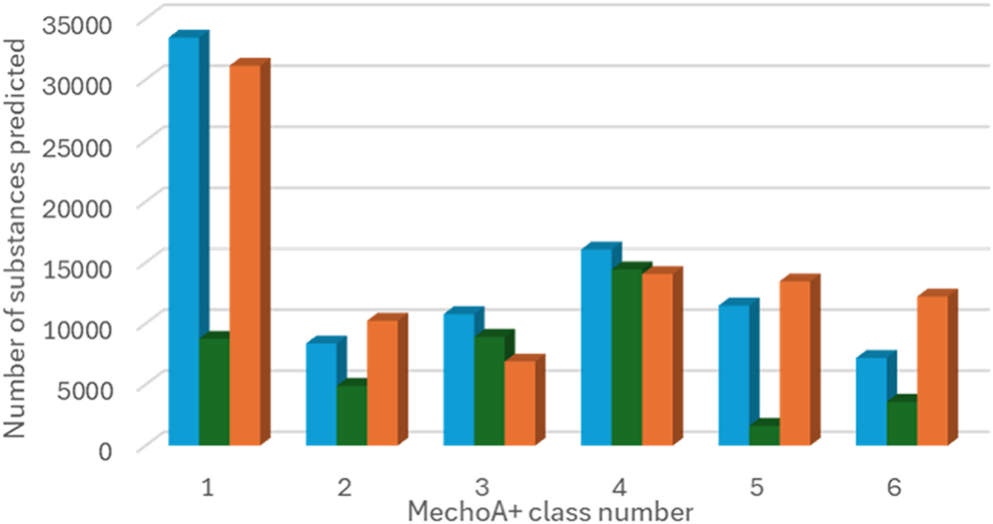
Number of substances predicted in each class using MechoA+ (light blue), SF (green), and MechoA (orange) schemes. Multiple occurrences of the same class number in a single prediction were counted as a single occurrence.

CLASS 1: the majority of the predictions of MechoA+ fall within “membrane destabilization” (narcosis). As discussed by Ellison et al.^13^ and Firman et al.,^22^ for a test set representative of the chemical space currently available, it is not surprising. Indeed, all chemicals will elicit baseline toxicity at an acute level in addition to any toxicity at lower concentrations resulting from more specific/receptor-mediated mechanisms, if they are present.^46^

CLASS 2: MechoA+ distinguishes whether the hydrolysis products will lead to nonpolar narcosis, polar narcosis, or to other mechanisms. The refinement of the class 2, usually called “ester narcosis,” leads to new alert definitions in MechoA+ scheme. The number of predictions was increased compared to SF scheme and decreased compared to MechoA scheme. The latest is explained by the restriction at the level of alert 151 for “ester, phosphate, carbonate, or carbamate” refining the scope of the alert.

CLASS 3: MechoA+ identifies more substances having “spontaneous reactivity” compared to the previous schemes. Indeed, SF has a lot of alerts for reactivity (coming mostly from Enoch et al.24) that MechoA did not completely cover, allowing it to encompass a broader range of substances.

CLASS 4: predictions about a metabolic first step cover about a quarter of the overall predictions, showing the importance of metabolism information for many chemicals. Additional work would be needed in this area since the exact identity of expected transformation products or metabolic pathways is not provided by the tool.

CLASS 5: the “Indirect biological systems disruption” is the third most represented class, highlighting the new restrictions implemented (notably for MechoA 5.2) compared to the MechoA scheme and also the new structural alerts compiled from newer experimental and mechanistic knowledge.

CLASS 6: The MechoA+ scheme predicts fewer substances with ″specific interaction with endogenous macromolecules″ mechanisms compared to MechoA, but twice as many as the SF scheme, resulting from the additional mechanisms introduced from the SF scheme. However, the decrease compared to MechoA is explained mostly by an overly predicted general alert in the MechoA scheme which was leading to overconservative predictions.

Not included in this analysis, the taxa applicability work performed during this project has particularly refined the scope of the MechoA 6 alerts. On another note, the necessity to cover more specific mechanisms, especially those concerning pharmaceutic agents, and to better identify potential endocrine active substances (EAS) has been identified. A set of new rules to predict potential endocrine modalities is already under development by the authors.

### 3.10 Structural Coverage Improvement

A t-SNE approach was used to plot the chemical space in 2D based on the same test set. The coverage of each scheme is presented in Figure 8.

**Figure 8:**
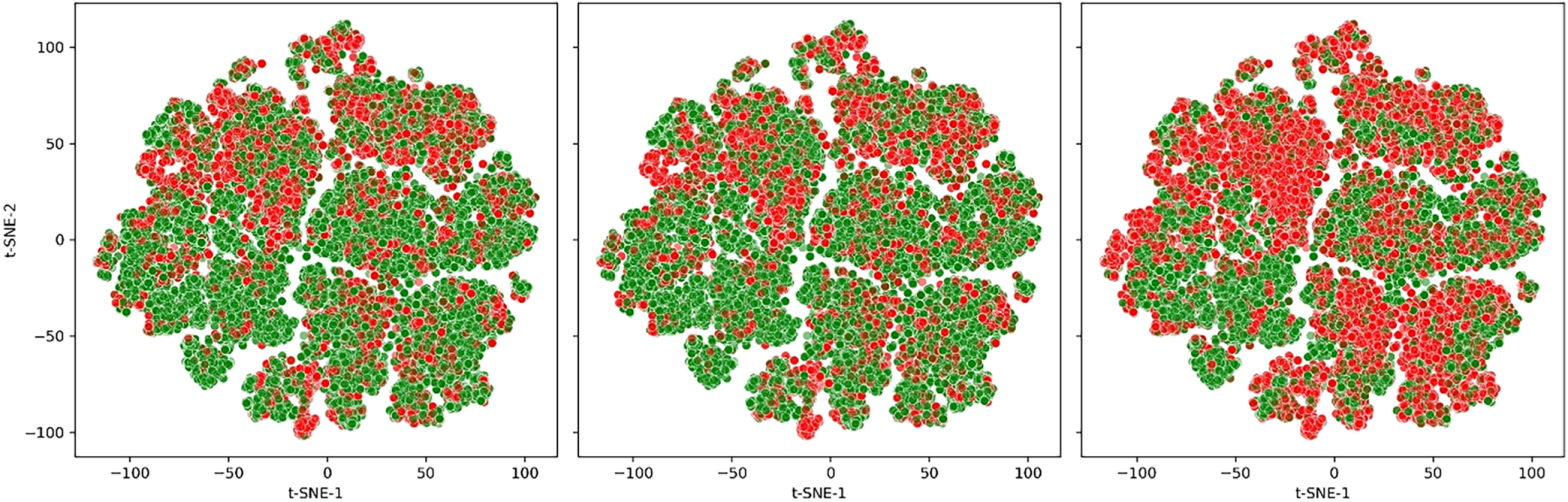
t-SNE visualization of the predicted versus not predicted substances for MechoA+ (left), MechoA (middle), and SF (right) schemes (green: predicted substances, red: not predicted substances).

This visualization shows the relevance of merging the former schemes into a new one since the MechoA+ scheme has a wider structural coverage compared to the former schemes (more green than red). In the end, MechoA+ better encompasses the chemical structural variety that (eco)­toxicologists may come across during their daily life, and it has a larger and better-defined coverage than any of the previous MIE-based models.

### 3.11 Use of MechoA+

MechoA+ offers a more comprehensive and accurate tool for evaluating the mechanisms of action of substances across diverse species. It represents a significant advancement in the understanding of the mechanistic basis of chemical toxicity, offering a valuable addition to the (eco)­toxicologist’s toolkit.

Building on the strengths of the tool, MechoA+ has a broad range of uses. It is automated and user-friendly with its “wheel” like visual and short text outputs. Since the results are MIE-based, they may be aligned to AOPs and therefore support toxicological interpretation. On top of that, MechoA+ unifies toxicologists and ecotoxicologist alike since a broad range of species can be associated with each MIE predictions.

The profiler is a tool that can help in reducing, refining, and replacing animal testing providing mechanistic insight. The results can support read-across strategies (including adhering to ECHA’s Read Across Assessment Framework, RAAF (Supporting Assessment Element 2.2)) or grouping argumentation.

Successfully implemented within an internal version of iSafeRat Desktop, it is also available as an add-in of the OECD QSAR Toolbox,^29^ ensuring its widespread adoption and accessibility for use in both regulatory and research applications. Performing an analogue search to find mechanistically related molecules is possible and is a powerful addition to existing profilers. Furthermore, the developers are currently working on an API for Online access to MechoA+. Besides, using MechoA+ in the first steps of the conception of new chemical products can help guide the development of safer alternatives, anticipate compliance, and reduce late-stage redesign.

Additionally, this profiler can be used as a building block to develop mechanistic QSARs meeting the OECD principles of validity, especially the fifth, which is mechanistic interpretation. As examples, acute and chronic ecotoxicity models developed for algae, daphnid, and fish meeting the criteria of OECD Guidelines, and developed using MechoA+ classification rather than structure (i.e., quantitative mechanism-activity relationships or QMARs), are already available.^30^

MechoA+ can be useful for many toxicological end points, such as skin sensitization, in vitro mutagenicity, or acute and chronic fish toxicity; however, MechoA+ does not provide quantitative hazard information as a standalone tool. Depending on the end point of interest, the user will need additional information to quantify or eliminate the potential for hazardous effects. Information on physicochemical parameters, additional toxicokinetic insight (absorption, distribution, metabolism, and excretion properties), and information on autoxidation or skin permeability can be used to quantify certain human health parameters. For instance, log *K*_OW_ (or alternatives such as membrane-water partitioning (K_MW_))^47–49^ or water solubility and results from quality ecotoxicity studies would be necessary to develop quantitative ecotoxicity models. MechoA+ requires further work to make it a useful quantitative predictor for end points such as carcinogenicity, developmental and reproductive toxicity, or repeated dose toxicity.

To address one part of this issue, an endocrine modality profiler is currently under development and will be implemented in MechoA+ in the near future.

Overall, within a single framework, MechoA+ has shown greater mechanistic, structural, and taxonomic domain coverage than existing schemes, with its ability to predict MIEs across a wide range of species. Its wide coverage, structured, and interpretable outputs, formulated in concise sentences, make it suitable for both high-throughput screening and mechanistic analysis.

## Supplementary Material





## Notes

Supporting Information 1 (3 Figures and 12 Tables) (XLSX)
Supporting Information 2 (4 Notes) (PDF)

## Abbreviations

MechoA: mechanism of toxic action
MoA: mode of action
QSAR: quantitative structure–activity relationship
REACH: regulation evaluation authorisation and restriction of chemicals
PBT: persistence, bioaccumulation and toxicity
NAMs: new approach methodologies
3R: replace, reduce, refine
AOP: adverse outcome pathway
MIE: molecular initiating event
SSbD: safe and sustainable by design
SAR: structure–activity relationships
SF: Sapounidou-Firman
SMARTS: SMILES Arbitrary Target Specification
iSafeRat: *in Silico* algorithms for environmental Risk and toxicity
t-SNE: t-distributed stochastic neighbor embedding
TP: true positives
FP: false positives
AD: applicability domain
ETC: electron transport chain
EAS: endocrine active substances
RAAF: read across assessment framework
QMARs: quantitative mechanism-activity relationships
