# Supplementary material for: MechoA+: A Chemical Structure Profiler Raising the Bar for the Prediction of Mechanisms of Toxic Action for Chemical Safety Assessment
Source: Environ Sci Technol. 2026 Jun 8;60(24):17124–35. doi: 10.1021/acs.est.5c18657 (PMC13296493; doi:10.1021/acs.est.5c18657)
Supplement: Supplementary file 2 [file es5c18657_si_002.pdf]

Supporting information 2

Title: MechoA+: a chemical structure profiler raising the bar for prediction of mechanisms of toxic action for chemical safety assessment

Gaspard Levet[a], Franklin J. Bauer[a], Paul C. Thomas[a], Mark T.D. Cronin[b], Jayne Roberts[c], Steve Gutsell[c], Bruno Campos[c], Geoff Hodges[c], James Firman[b]*

[a]KREATiS SAS, 23 rue du Creuzat, ZAC de St-Hubert, 38080 L'Isle d'Abeau, France

[b]School of Pharmacy and Biomolecular Sciences, Liverpool John Moores University, Byrom Street, Liverpool, L3 3AF, UK

[c]Safety, Environmental and Regulatory Science (SERS), Unilever, Colworth Science Park, Sharnbrook, Bedfordshire MK44 1LQ, UK

Supporting information 2 consists in 9 pages with 4 Notes, 1 Additional references section.

Note S1. Taxa extrapolation for specific MechoAs

The “traditional” method is to derive the taxonomic applicability from literature-based species applicability and then extrapolate the applicability of the MIE to other species.

However, for alerts related to specific mechanisms that act on a specific biological target in an organism (e.g. receptors, ion channels, etc.), the taxonomic applicability was refined by using the protein ortholog database EggNOG v5.0.0 [1] and Taxonomy Browser from NCBI [2]. It was decided to consider only the taxa which have the same protein/gene identifier as the MIE target (e.g. “AChE” for acetylcholinesterase or “CYP19A1” for aromatase), without further evaluating the protein similarity. The number of species expressing the target protein is given by EggNOG. Thereafter, a batch list of the taxonomical identifiers can be run through NCBI Taxonomy Browser Common Tree [2] to visualize the taxonomy tree of a set of species which were attributed to the MIE.

Note S2. Prediction results formatting

MechoA+ predictions are given as follows:

MechoA xxY.Z: Sentence describing the MIE with an indication of the taxa concerned.

- “xx” is the taxa code relating to the taxonomic applicability of the MIE,

In the case if taxa applicability is “for all species”, i.e., all species may be impacted by such MIE, “xx” is absent. For example, in the case of the alert about acrylates, the model would predict “MechoA 3.2: Michaël addition with sulfhydryl groups, generating protein and DNA adducts for all species.”

- “Y” the class
- “Z” the sub-class
- ‘C’ can appear between the prefix “xx” and the class number “Y.Z”, to designate that the toxicological outcomes of the mechanism of action can be observed in chronic timeframe only (e.g. carcinogenicity).
- Separation characters
    - “&” = “And” (the following MechoA is relatively certain). It was needed to separate predictions when several MIEs are predicted for a substance.
    - “/” indicate a degree of uncertainty of the results. This would be the case when the MIE leading to observed toxicological outcomes is not completely understood. For instance, this is the predicted MechoA for propham derivatives: “MechoA 6.2 / pl6.7: disruption of calcium transport in all species and inhibition of tubulin polymerisation into microtubules, which may be a direct inhibition or simply be due to the modified calcium concentration, stopping mitosis in plants.”

In the new scheme, standardisation of the taxa code into a two-letter code was performed. New terms and abbreviations were needed compared to MechoA scheme v2.2 (available in OECD QSAR Toolbox) to better characterize the taxa (03), while some too wide, unspecific, polyphyletic / paraphyletic groups, such as “unicellular organisms” were removed.

Table 13. MechoA+ taxa codes attribution

!	for all species but this one

pr	MechoA for procaryotes
ba	MechoA for bacteria
eu	MechoA for eucaryotes
an	MechoA for animals (i.e. Metazoa)
vr	MechoA for vertebrates
ma	MechoA for mammals
pt	MechoA for primates
fi	MechoA for fish (i.e. Teleostei)
bi	MechoA for birds (i.e. Aves)
ps	MechoA for Protostomia (i.e. the family including most invertebrate animals, like arthropods, molluscs, annelids, flatworms and nematodes)
ar	MechoA for arthropods
pl	MechoA for plants (see below *)
fu	MechoA for fungi

* The term "pl" for "plants" in this scheme refers to all photosynthesizing organisms, thus including but not limited to: viridiplantae, cyanobacteria, glaucophytes, cryptophytes, haptophytes, dinoflagellates, chromerids, ochrophytes, chlorarachniophytes, euglenids, some cnidarians, some sponges, some aquatic gastropods, some marine flatworms.

After the MechoA code, a text clarifies and provides details on the predicted mechanism, applicable species. This text also often describes further key events typically occurring from the predicted MIE. More information is provided with the web page of MechoApedia which is in the process of being updated to provide more information on the MIE described for each alert and class.

Note S3. Correction of identified issues related to MechoA scheme and its implementation

The authors noticed four redundant issues when they worked on the improvement of the performance of the model after definition of the alerts.

The first issue observed was in the reading of SMILES. iSafeRat® Desktop code has some restrictions on the usable SMILES (detailed in the user guide of iSafeRat® Desktop). For instance, SMILES codes for mixtures of organic constituents cannot be processed by the software. iSafeRat® Desktop also needs to transform some SMILES as a standardisation step, rewriting aromatic cycles using lowercase letters. But this transformation is not always workable in the current version. Also it was observed that tautomer forms could not be taken into account by the software. If a single molecule has a tautomer, it could be identified as two separate molecules leading to two separate predictions. Thus, in those particular cases, an expert judgment would be needed to run the prediction on the most favourable tautomer form.

The second issue concerned SMARTS implemented in the alerts. Certain SMARTS originally failed to trigger relevant alerts. The SMARTS language was well described by Daylight [3], and it is expected that there is no distinction between tools when reading a string. However, it was noticed that, depending on the tool, identical SMARTS could be read differently (e.g., SMARTS PLUS [4] compared to RDKit library [5] as implemented in iSafeRat® Desktop). Contrary to our expectations, some means of writing SMARTS, relative to other theoretically equivalent renderings, could lead to different results. Since there are usually several means of writing SMARTS to describe one pattern, all identified problematic patterns could be successfully corrected.

The third was about the structural patterns specified directly as C++ code using the molecular connectivity matrix calculated by the software without the use of RDKit or SMARTS strings. In these “C++ code blocks”, some minor bugs in the implementation were noticed and corrected. Restriction in the SMARTS or C++ code contributed in the definition of the scope of the alerts.

The fourth was about the decision tree. Using the training set developed for the model, the authors noticed that some of the alerts were “hidden” because of the prioritisation rules. That is, the alert was never read, because the molecules corresponding to this alert were matched by other alerts higher in the decision tree, that are a “stop” for the model. This issue arises when alerts encompass a large number of molecules. Indeed, such alerts often have less restrictive SMARTS and if placed before more restricted alerts targeting similar molecules, they will mask the prediction for these given alerts. It was chosen to keep as many alerts as “stop” as possible to maintain the goal of the decision tree to produce simple outcomes, presenting only the most impactful MIEs. Depending on the case, this problem was overcome by two ways: either by upgrading the position of the hidden alert above the hiding alert in the decision tree, or by restricting the structural domain of the hiding alert. For instance, it was the case for the alert 4 about carbamates which was hiding the alert for propham derivatives. In this particular case, it was better to rework the structural domain of the alert 4 than to interchange the relative positions of the two alerts because the alert 4 detects neurotoxic carbamates which was judged of higher priority than propham alert (associated with herbicidal activity) in the scheme.

Note S4. Explanation of the results of the column in the training set

To build the training set of 2091 substances available in the Supporting Information 1 Table 3, the authors gathered the SMILES and when easily available, the CID number (pubchem number), the name and the CAS number of the substances. To each of them, an alert number or several alert numbers were associated ("MechoA+_rule_number" column). Then, the associated mechanism expected for each substance as well as their predicted MechoA is underlined ("MechoA expected from this alert" column and "MechoA+_prediction_v4.3.46" column) with some experimental evidence based on studies or associated references ("Observed mechanisms and effects" column and "Reference" column).

Four columns were needed to analyse all the information included in this dataset and account for the variability and uncertainty of the results:

- "Experimental alert validity" column: i.e. does the experimental/literature evidence match with the MechoA expected from the alert? The conclusion for each substance is synthesised in 5 simple answers:
    - "valid" *i.e.* MechoA from the alert(s) matches experimental evidence,
    - "partially valid"
        - insufficient evidence available to confirm the overall MechoA for a substance,
        - only a part of the MechoA is wrongly affiliated according to experimental evidence
    - "unknown" *i.e.* not sufficient evidence is available to confirm the MechoA for a substance,

- “invalid” *i.e.* sufficient evidence is available to reject the expected MechoA for a substance,
- “not invalid” *i.e.* sufficient evidence are available to confirm that the expected MechoA should not be triggered for a substance.

- “Prediction alert validity” column: is the substance predicted at least according to the alert? Is the substance hit at least by this expected alert (but can be hit by other alerts too)? This column distinguishes if a structure matched by the alerts corresponds to the expected definition of the structural alert described in SMARTS code or C++ code. The conclusion is synthesised in 4 answers:
  - “valid” *i.e.* alert is triggered as it should be (equivalent to “True positive”),
  - “partially valid”: account for the uncertainty in the results which could come from several cases:
    - lack of experimental evidence about the overall MechoA prediction does not give enough confidence to confirm the overall result,
    - several MIE are identified for a given substance but one or several MIE identified experimentally are missing
    - only a part of the alert is triggered as it should be, a part of the MechoA prediction is wrong, for example when a substance trigger two alerts, one is valid based on experimental evidence while the other is invalid for the given substance

        - another relevant alert could have been triggered, but because of the decision tree it is not
    - "invalid" *i.e.* substance identified in the alert are not predicted as they should be compared to the definition of the structural alert in the model
    - "not invalid" *i.e.* alert may be triggered as it should be but the reference substance has no data or is in the dataset only to prove true negative results
- "Validity of each alert prediction" column: i.e. is the substance predicted according to the experimental/literature evidence for this alert? If several alerts are triggered for a substance (when there is species differences), the results of each "Prediction alert validity" is taken into account to make a consensus result
- "Overall validity MechoA+" i.e. does the prediction match the experimental/literature evidence, all alerts being considered? It compares the experimental results with the prediction, based on "Experimental alert validity" and "Validity of each alert prediction" columns. Taking into account all cases, based on the former responses in both columns, 6 results might be possible:
    - Valid (equivalent to true positive (TP))
    - Valid a priori (equivalent to TP with a degree of uncertainty)
    - Partially valid (not possible to distinguish if it is a TP or FP at the moment)
    - Partially valid a priori (not possible to distinguish if it is a TP or FP at the moment)
    - Invalid (equivalent to false positive (FP))
    - Invalid a priori (equivalent to FP with a degree of uncertainty)

Note S5. Comparison of ecotoxicity results

A comparison of MechoA predictions generated by iSafeRat® between MechoA+ and MechoA Premium (on which all the ecotoxicity QSARs are built) found only 2 out of 462 substances with a different MechoA prediction for sub-classes linked to QSARs.

Additional references

[1] J. Huerta-Cepas, D. Szklarczyk, D. Heller, A. Hernández-Plaza, S.K. Forslund, H. Cook, D.R. Mende, I. Letunic, T. Rattei, L.J. Jensen, C. von Mering, P. Bork, eggNOG 5.0: a hierarchical, functionally and phylogenetically annotated orthology resource based on 5090 organisms and 2502 viruses, Nucleic Acids Research 47 (2019) D309–D314. https://doi.org/10.1093/nar/gky1085.

[2] Common Taxonomy Tree, (n.d.). https://www.ncbi.nlm.nih.gov/Taxonomy/CommonTree/wwwcmt.cgi (accessed August 11, 2023).

[3] Daylight Theory: SMARTS - A Language for Describing Molecular Patterns, (n.d.). https://www.daylight.com/dayhtml/doc/theory/theory.smarts.html (accessed August 2, 2023).

[4] SMARTS.plus, (n.d.). https://smarts.plus/ (accessed August 22, 2023).

[5] RDKit, (n.d.). https://www.rdkit.org/ (accessed August 22, 2023).
